# Chemical, Manufacturing, and Standardization Controls of Grape Polyphenol Dietary Supplements in Support of a Clinical Study: Mass Uniformity, Polyphenol Dosage, and Profiles

**DOI:** 10.3389/fnut.2021.780226

**Published:** 2021-12-16

**Authors:** Weiting Lyu, David Rodriguez, Mario G. Ferruzzi, Giulio M. Pasinetti, James W. Murrough, James E. Simon, Qingli Wu

**Affiliations:** ^1^New Use Agriculture and Natural Plant Products Program, Department of Plant Biology and Center for Agricultural Food Ecosystems, Institute of Food, Nutrition & Health, Rutgers University, New Brunswick, NJ, United States; ^2^Department of Medicinal Chemistry, Ernest Mario School of Pharmacy, Rutgers University, Piscataway, NJ, United States; ^3^Independent Researcher, Carlstadt, NJ, United States; ^4^Department of Food, Bioprocessing and Nutrition Sciences, Plants for Human Health Institute, North Carolina State University, Kannapolis, NC, United States; ^5^Department of Neurology, Icahn School of Medicine at Mount Sinai, New York, NY, United States; ^6^Geriatric Research, Education and Clinical Center, James J. Peters Veterans Affairs Medical Center, Bronx, NY, United States; ^7^Depression and Anxiety Center for Discovery and Treatment, Department of Psychiatry, Icahn School of Medicine at Mount Sinai, New York, NY, United States

**Keywords:** botanicals, quality control, grape seed extract (GSE), resveratrol, grape juice, LC/UV-Vis/MS, product quality, authentication

## Abstract

Bioactive dietary polyphenols in grape (*Vitis vinifera*) have been used in Dietary Supplements (DSs) with the aim to prevent numerous diseases, including cardiovascular and neurodegenerative diseases, and to reduce depression and anxiety. Given prior recognition that DSs can be quality challenged from the purity, authentication, adulteration, and actual concentration of targeted bioactives, to ensure consumer health protection as well as the quality and safety of grape polyphenol-based DSs, the present investigation was aimed at establishing a comprehensive quality control (QC) approach for grape polyphenol-based DSs in support of a human clinical study. In this study, the manufactured grape seed polyphenol extract (GSPE) and *trans*-resveratrol (RSV) capsules and Concord Grape Juice (CGJ) along with the corresponding original drug materials were analyzed using the developed different liquid chromatography/UV-visible spectroscopy/mass spectrometry (LC/UV-Vis/MS) methods. The weight variation of GSPE and RSV capsules was also evaluated according to the US Pharmacopeia (USP) tests. The results indicate that the total identified polyphenol content in each grape seed extract (GSE) capsule/CGJ is very similar and all GSE/RSV capsules pass the content/weight uniformity test. Given the complexity of these and many botanical products from the issues of purity, quality, adulteration, consistency, and their coupling to the complex chemistry in each grape-derived botanical, quality assurance and the steps needed to ensure grape-derived DSs being well homogeneous and stable and containing the known and expected bioactives at specific concentration ranges are fundamental to any research study and in particular to a clinical trial. Each of these issues is essential to provide a solid foundation upon which clinical trials with botanicals can be conducted with the goal of realizing measurable mental health outcomes such as reducing depression and anxiety as well as understanding of their underlying biological mechanisms.

## Introduction

Consumer interest in the use of botanical dietary supplements (DSs) continues to increase. The global DS marketplace was valued at USD 132.8 billion in 2016 and expected to reach USD 220.3 billion in 2022 (1). In particular, the US ranks as the leading country in botanical DS consumption. It is estimated that 77% of US adults consume DSs on a regular basis (2). The consumer demand and growth in the DS industry reinforce the importance of ensuring safe and high-quality DS products.

*Vitis vinifera* (grape) is one of the most widely cultivated fruit species in the world, and the total production of grapes worldwide is ~60 million tons (3). Grape and grape-derived products contain a unique mixture of bioactive dietary polyphenols, which have long been reported to have antioxidant and positive health promoting effects and associated with the prevention of numerous diseases, including cardiovascular and neurodegenerative diseases as well as several forms of cancers (4–6). Previous studies have investigated the disease preventative effect of some specific grape polyphenol forms, including resveratrol (RSV), proanthocyanidins, and anthocyanins (7–9). Thus, grapes and their byproducts are the ideal candidates for DSs.

Most grape polyphenols can be found in grape juice after an extraction through pressing, and Concord Grape Juice (CGJ) is one of the main processed products of grapes. Grape seed is also one of the major industrial byproduct of the winemaking process, and more than 70% of grape phenolics can be retained in skins and seeds (10). Therefore, grape seed extract (GSE) is a popular and widely used DS in the USA. *Trans*-RSV (systematically termed as *trans*-3,5,4′-trihydroxystilbene), which is produced by grape berries of *Vitis* varieties in response to UV irradiation, is an antioxidant compound found in the skin of grapes (11). The potential role of RSV in health promotion, such as the prevention and treatment of diabetes, cancer, obesity, pain, inflammation, tissue damage, and even “aging,” has made it increasingly popular in recent years as a DS (12).

However, the adulteration of grape-derived botanical products can also be a significant problem. In a study, using liquid chromatography–mass spectrometry (LC-MS) and thin layer chromatography (TLC), researchers found that of 21 commercial GSE products 6 were adulterated and might contain allergens, notably peanut skins (13). Because consumers rely on label claims and other information that are provided directly from the supplier, the adulteration of those DSs, especially with a common allergen, represents a considerable risk to public safety. Therefore, more sophisticated and proper analytical tests are needed to detect such adulteration.

Chemistry, Manufacturing, and Controls (CMC) is an integral part of any pharmaceutical product application to FDA. There is an intrinsic link between the CMC attributes of a pharmaceutical product and the safety and efficacy of clinical therapy (14). The US Pharmacopeia (USP) and the National Formulary (NF) drug substance and excipient monographs, as well as general tests and procedures, are frequently cited in New Drug Applications (NDA) (15), and a summary of pharmaceutical test scheme for pharmaceuticals and DSs is presented in Table 1. Because of the coupling of CMC to the recognition that some commercial botanical products on the marketplace were quality challenged, to ensure consumer health protection as well as the quality and safety of grape-based DSs, the present research was aimed at establishing a clear and comprehensive quality control (QC) approach for the grape-based DS that would be used for clinical trials. In this study, GSE and RSV capsules and CGJ were analyzed using our optimized high-performance liquid chromatography coupled with UV coupled with electrospray ionization tandem mass spectrometry (HPLC-UV/Vis-MS) and ultra-high-performance liquid chromatography coupled to triple quadrupole mass spectrometry (UHPLC-QQQ/MS) methods. The weight variation of GSE and RSV capsules was also evaluated according to the USP tests.

**Table 1:** Pharmaceutical test scheme for pharmaceuticals and dietary supplements (DSs).

**Pharmaceuticals**	**Dietary supplements**
<301> Acid-neutralizing capacity	<1216> Tablet friability
<701> Disintegration	<2040> Disintegration and dissolution of dietary supplements
<711> Dissolution	<2091> Weight variation of dietary supplements
<724> Drug release	<2750> Manufacturing practices of dietary supplements
<785> Osmolarity	
<905> Uniformity of dosage forms	
<1087> Apparent intrinsic dissolution—dissolution testing procedures for rotating disk and stationary disk	
<1088> *In vitro* and *in vivo* dissolution evaluation of dosage forms	
<1090> Assessment of drug product performance—bioavailability, bioequivalence, and dissolution	
<1216> Tablet friability	

*Note: Numbers in angular brackets refer to chapter numbers in the General Chapters section from US Pharmacopeia (USP) 41 and National Formulary (NF) 36*.

## Materials and Methods

### Chemical Reagents

US Pharmacopeia reference standard compounds, including *trans*-RSV, (+)-catechin, (–)-epicatechin, gallic acid, 3-hydroxytyrosol, isochlorogenic acid, 3, 4-dihydroxyphenylacetic acid, 4-methyl gallic acid, 3, 4-dihydroxyphenylacetic acid, 3-hydroxybenzoic acid, caffeic acid, 4-hydroxybenzoic acid, vanillic acid, dihydromyricetin, syringic acid, resveratrol-3-glucoside, dihydroferulic acid, sinapic acid, taxifolin, ferulic acid, 3-hydrocinnamic acid, phenylacetic acid, and *trans*-2-hydrocinnamic acid, were purchased from Sigma Chemical Co. (St. Louis, MO, USA). Primary analytical standard (Grade P) compounds, including procyanidin B2, procyanidin C1, quercetin, and cyanidin-3-glucoside, were purchased from ChromaDex (Irvine, CA, USA). The HPLC grade water, acetonitrile (ACN), methanol (MeOH), formic acid (FA), and trifluoroacetic acid (TFA) were obtained from Fisher Scientific Co. (Fair Lawn, NJ, USA).

### Drug Material Sourcing

Three kinds of grape-based DSs were analyzed.

MegaNatural® grape seed polyphenol extract (GSPE) was purchased from Polyphenolics Company (Madera, CA, USA). The grapes were grown in California, USA and certified by Halal (IFANCA), and the final GSPE product was processed using hot water extraction at a ratio of 30:1 or −50:1 (dry seed: extract).

Synthetic *trans*-RSV was purchased from BannerBio Nutraceuticals, Inc. (Nanshan District, Shenzhen, China).

The CGJ concentrate was provided by Welch Foods, Inc. (Westfield, NY, USA). The CGJ concentrate was squeezed from Concord grapes grown and harvested in the Eastern USA growing region and processed in Westfield, NY, USA, within 8 h of harvest, and were pasteurized to achieve a 5-log pathogen reduction and commercial sterility.

### Manufacturing of GSPE and RSV Capsules and CGJ

Original GSPE and RSV materials were delivered to Eagle Nutritionals (Carlstadt, NJ, USA) to manufacture the final products using encapsulation at specific concentrations required for planned clinical trials in a NIH-funded U19 study. Briefly, for GSPE capsules, 450 mg of GSPE powders and 50 mg of silica were filled into #0 purple/white hard gelatin capsules. For RSV capsules, three different weight levels (150, 300, and 450 mg) of RSV powders were encapsuled using #0 green capsules.

**Figure 1:**
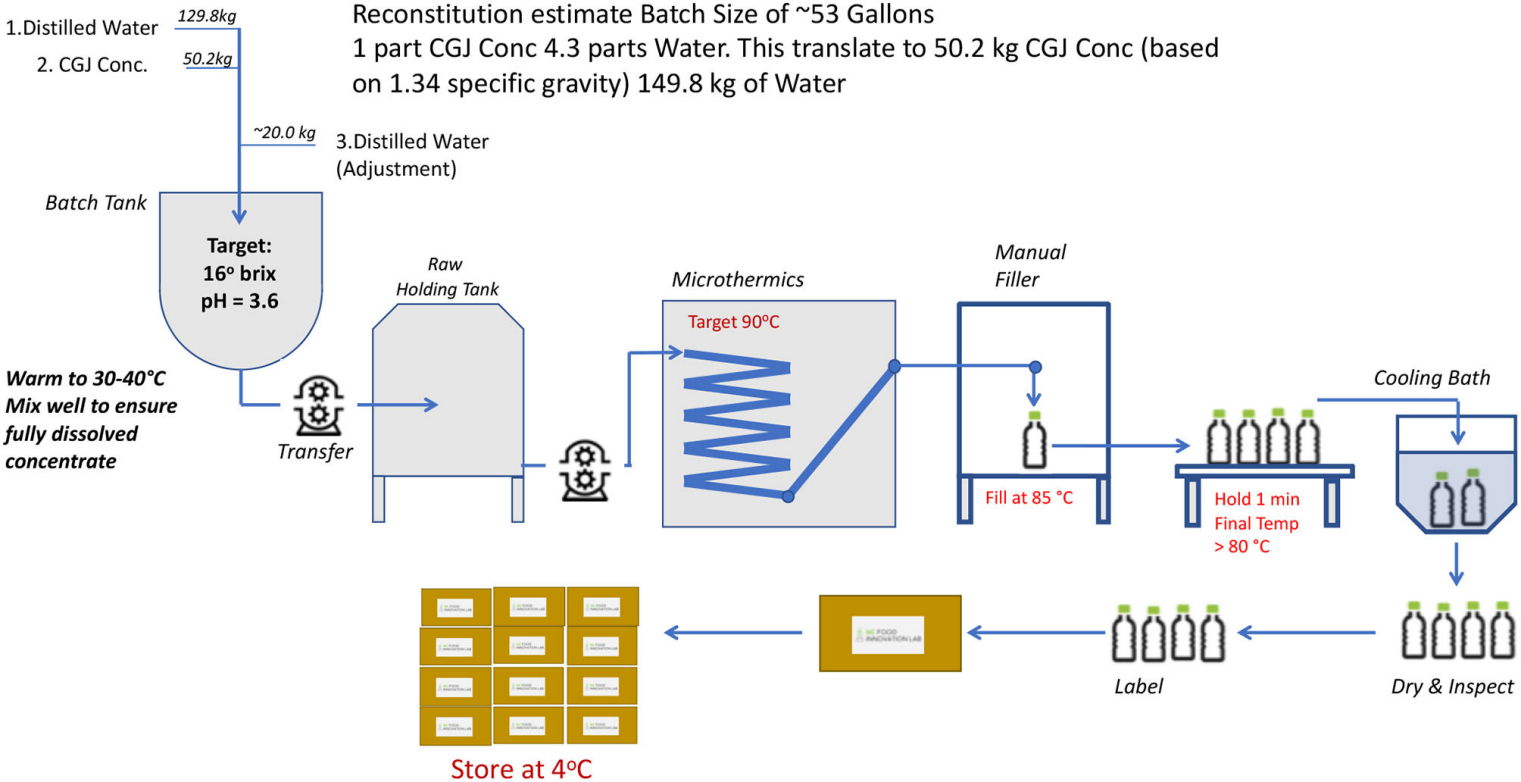
Concord Grape Juice (CGJ) processing flow.

Concord Grape Juice is reconstituted from the CGJ concentrate to single strength 100% CGJ. The general process flow was shown in Figure 1. Briefly, 129.8 kg of distilled water was transferred into a 50-gallon batch tank and warmed up to room temperature, and 50.2 kg of the CGJ concentrate was added to the tank. The mixer was warmed to 30°C and gently mixed for 10–15 min to ensure proper mixing. A mixer sample was analyzed for pH and adjusted with remaining water as needed to achieve the target of 16.1°brix and to achieve and confirm the final pH of 3.5–3.7. The final product was transferred to an original product holding tank in a thermalization room adjacent to the Microthermics. Finally, the CGJ was hot filled into 8 oz PET bottles, and following cooling to <40°C bottles it was removed from a bath, dried, and inspected.

### QC of Original Drug Materials of GSPE, RSV, and CGJ Concentrate

#### QC of Original GSPE Materials

The container and inside package of the GSPE original material product were opened and from five distinctly different physical locations subsampled for chemical profiling in Eagle Nutritionals using standard industry subsampling procedures. The five subsamples were carefully placed into ziplock plastic bags, labeled, and then transported to Rutgers University for chemical analysis in our lab. For each of the five subsamples, three replicates were made in parallel for the QC process. Approximately 30 mg of GSPE original material was accurately weighted and prepared in 10 ml 70% MeOH with 1% FA, vigorously vortexed, and sonicated for 10 min. An aliquot of 200 μl of the extract was diluted by mixing with 0.8 ml 70% MeOH with 1% FA and centrifuged at 16,000 rpm for 10 min, and then the supernatant was injected for the LC-UV/Vis-MS analysis.

For the preparation of reference solutions of gallic acid, (+)-catechin, (–)-epicatechin, procyanidin B2, and procyanidin C1, *ca*. 5 mg of each standard was accurately weighed and diluted to 10 ml using 70% MeOH with 1% FA. Each standard stock solution was sonicated for 10 min, and was allowed to cool down to room temperature. Next, 2 ml of each standard stock solution was allowed to combined together with each other and sonicated for 10 min to mix well to form a standard mixture of gallic acid, (+)-catechin, (–)-epicatechin, procyanidin B2, and procyanidin C1. Further serial dilutions up to 100~0.1 μg/ml were made using the same solvent.

An Agilent 1290 Infinity II UHPLC (Agilent Technology, Palo Alto, CA, USA) equipped with a diode array detector (DAD) and 6546 quadrupole time-of-flight (Q-TOF) MS with electrospray ionization source (ESI) (Santa Clara, CA, USA) was used for chromatographic separation. Nitrogen generated from the Parker Balston NitroFlow60NA nitrogen generator was used for MS electrospray ionization. MassHunter Workstation software Data Acquisition (version B.08.00) was used for data processing. An Agilent Polaris Amide-C18 (250 × 4.6 mm, 3 μm) column was used for compound separation. For the LC part, the mobile phase A was water with 0.1% FA, and mobile phase B was ACN with 0.1% FA. The gradient was 2% B at 0 min and held for 3 min, raised to 15% B at 15 min and held until 25 min, then raised to 35% B at 50 min, 60% B at 51 min, and held until 55 min. The column was equilibrated with 2% B for 3 min between injections. The flow rate was 0.8 ml/min. The column was set at 40°C, and an autosampler was maintained at 4°C. The injection volume was 2.5 μl. A diode array detector (DAD) was set at 280 nm, with the bandwidth at 4 nm. The reference wavelength was 400 nm, with the reference bandwidth at 10 nm. For the MS condition, the gas was dried at 300°C with a flow rate of 12 L/min. Sheath gas was dried at 250°C with a flow rate of 12 L/min. The nebulizer pressure was 30 psi. The VCap was 4,000 V, and the nozzle voltage was 500 V. Fragmentor voltage was set at 180 V, skimmer was 65 V, and Oct 1 RF Vpp was 750 V. The scan ranged from 150 to 1,700 m/z. Acquisition rate was 6 spectra/s.

Gallic acid, catechin, and epicatechin were quantified with the calibration curve of corresponding reference standards. Procyanidin dimers (P_2_) were quantified based on the calibration curve of procyanidin B2, and procyanidin trimers (P_3_) were quantified based on the calibration curve of procyanidin C1 as shown in Supplementary Table S1.

#### QC of Original RSV Materials

The container and inside package of the RSV original material product were opened and from three different physical locations subsampled for chemical profiling in Eagle Nutritionals. Reference standard and RSV samples were prepared under dark conditions, and opaque Falcon® tubes and brown Eppendorf® tubes were used. For each of the three subsamples, three replicates were made in parallel for the QC process. *ca*. 50 mg was accurately weighed and dissolved in 10 ml 70% MeOH with 1% FA. An aliquot of 100 μl of the extract was diluted by mixing with 9.9 ml 70% MeOH with 1% FA, and then 100 μl of each diluted sample was further diluted to 1 ml with the same solvent. The solution was centrifuged at 12,000 rpm for 5 min, and then an aliquot of 2.5 μl of the supernatant was injected into UHPLC for analysis.

For the preparation of reference solutions of *trans*-RSV, *ca*. 5 mg of the standard was accurately weighed and diluted to 10 ml using 70% MeOH with 1% FA. The stock solution was sonicated for 10 min and was allowed to cool down to room temperature. Further serial dilutions up to 200~0.1 μg/ml were made using the same solvent.

Agilent 1290 Infinity II UHPLC equipped with DAD and 6546 Q-TOF MS with ESI were used for chromatographic separation. The column used for the RSV QC test was Kinetex™ (Torrance, CA 90501-1430 USA) C_18_ column (CO, USA), the particle size was 2.6 μm, and the size was 100 × 2.1 mm. For the LC condition, water with 0.1% FA was used as mobile phase A, and ACN with 0.1% FA was used as mobile phase B. The gradient was 25% B at 0 min, then raised to 60% B at 4 min, held until 4.5 min, then dropped to 25% B at 5 min. The flow rate was 0.35 ml/min, and the column was equilibrated with 25% B for 1 min between injections. The column was set at 40°C, and an autosampler was maintained at 4°C. The injection volume was 2.5 μl. The DAD was set at 210 nm (as the wavelength for general impurities), 280 nm (as the absorption maximum of *trans*-RSV), and 305 nm (for the possible degradation product *cis*-RSV). *Trans*-RSV in the RSV capsule was quantified based on the reference standard calibration curve under 280 nm. The calibration curve parameters are presented in Supplementary Table S1.

#### QC of CGJ Concentrate

##### Determination of Anthocyanidins and Flavonols Using LC-UV/Vis-MS

Three replicates of the CGJ concentrate were made in parallel for the QC process. To prepare the CGJ concentrate, 2.5 ml of the CGJ concentrate was diluted in 7.5 ml water. All samples were centrifugated at 12,000 rpm for 10 min, and the supernatant was directly injected into UHPLC. For the preparation of reference solutions of cyanidin-3-glucoside and quercetin, *ca*. 10 mg of each standard was accurately weighed and diluted up to 10 ml using 70% MeOH with 1% FA. Then, the standard stock solution was sonicated for 10 min and allowed to cool down up to room temperature. Afterward, 0.5 ml of all standard stock solutions were combined together and sonicated for 10 min to mix well to form a standard mixture. An aliquot of 200 μl stock solution was then spiked into 0.8 ml 70% MeOH with 1% FA to make the first working solution. Further serial dilutions of 100~0.1 μg/ml were made using the same solvent.

The Agilent 1290 Infinity II UHPLC equipped with DAD was used for chromatographic separation, and the column Agilent Polaris Amide-C18 (250 × 4.6 mm) was used for compound separation. The mobile phase A was water with 0.4% TFA, and B was ACN with 0.4% TFA. The flow rate was 0.8 ml/min. The gradient was 10–20% B from 0 to 20 min; 20–30% B from 20 to 35 min; isocratic elution at 30% B from 35 to 40 min; 30–60% from 40 to 50 min; and kept 60% from 50 to 55 min, then dropped to 10% B in 0.5 min. The column was equilibrated with 10% B for 2 min between injections. The column was set at 40°C, and an autosampler was maintained at 4°C. The injection volume was 2.5 μl. The DAD was set at 370 nm (as the absorption maximum of most flavonols) and 520 nm (as the absorption maximum of most anthocyanidins) with the bandwidth of 4 nm, and the reference wavelengths were set at 500 and 360 nm, respectively, with the reference bandwidth at 10 nm.

Anthocyanidins were quantified based on the calibration curve of cyanidin-3-glucoside at 520 nm, and the quantity was further adjusted based on the molecular weight ratio. Flavonols were quantified based on the calibration curve of quercetin at 370 nm, with a further adjustment of the quantity based on the corresponding molecular weight ratio. The calibration curve parameters are shown in Supplementary Table S1.

##### Determination of Other Phenolic Compounds Using UHPLC-QQQ/MS

To prepare the CGJ concentrate, 2.5 ml of the CGJ concentrate was diluted in 7.5 ml water. Then, the diluted CGJ concentrate samples were further diluted using 1% FA acidified 70% MeOH solution (1:10). The prepared samples were centrifugated at 12,000 rpm for 10 min, and the supernatant was directly injected into UHPLC. Three replicates were made in parallel for the QC process.

For the preparation of reference solutions of 3-hydroxytyrosol isochlorogenic acid, 3,4-dihydroxybenzoic acid, 4-methyl gallic acid, catechin, procyanidin B2, epicatechin, 3-hydroxybenzoic acid, caffeic acid, 4-hydroxybenzoic acid, vanillic acid, dihydromyricetin, syringic acid, resveratrol-3-glycoside, dihydroferulic acid, 3-hydroxycinnamic acid, taxifolin, sinapic acid, ferulic acid, phenylacetic acid, and *trans*-2-hydroxycinnamic acid, *ca*. 10 mg of each standard was accurately weighed and diluted to 10 ml using 70% MeOH with 1% FA. Each standard stock solution was sonicated for 10 min, and was allowed to cool down to room temperature. About 0.5 ml of each standard stock solution was combined together with each other and sonicated for 10 min to mix well to form a standard mixture. An aliquot of 100 μl stock solution was spiked into 10 ml 70% MeOH with 1% FA to make the first work solution. Further serial dilutions up to 5,000~0.1 ng/ml were made using the same solvent. For the preparation of samples, nine replicates were made in parallel for the QC process. All samples were diluted using 1% FA acidified 70% MeOH solution (1:10). The prepared samples were centrifugated at 16,000 rpm for 10 min, and the supernatant was directly injected into UHPLC.

The instrument used for chemical analysis was an Agilent 1290 Infinity II UHPLC (Agilent Technology, Palo Alto, CA, USA) hyphenated with 6470 triple quadrupole MS with ESI (Santa Clara, CA, USA). Agilent MassHunter Optimizer (version B.07.00) for standard compound-related parameter optimization and MassHunter Workstation software Data Acquisition (version B.08.00) and Quantitative Analysis (version B.07.01) for data processing were used. The column used for this section separation was an Agilent SB-AQ RRHD UHPLC column, the particle size was 1.8 μm, and the size was 150 × 2.1 mm with an SB-AQ guard column (2.1 × 5 mm, 1.8 μm). Nitrogen generated from Parker Balston NitroFlow60NA nitrogen generator was used for MS electrospray ionization. For the LC parameters, the mobile phase A was 0.1% FA in water, and mobile phase B was 0.1% FA in ACN. The flow rate was 0.2 ml/min, and the injection volume was 2.5 μl. The gradient was 4% B to 40% B in 6 min, and raised to 60% B from 6 to 10 min, then held at 60% B for 0.5 min, and dropped to 4% B in 0.5 min. The column was equilibrated with 4% B for 1 min between injections. The column was thermostatted at 30°C, and an autosampler was set to 4°C. Nitrogen was used as the nebulizing and drying gas. The nebulizer was set to 30 psi and the drying gas was set to 300°C with a flow rate of 13 L/min. The sheath gas was set to 250°C with a flow rate of 12 L/min. In the scan mode, dynamic multiple reactions of monitoring (dMRM) were optimized using MassHunter Optimizer as priorly reported, with the parameters presented in Table 2.

**Table 2:** The information for dynamic multiple reactions of monitoring (dMRM) parameters.

**Compound**	**Retention time (min)**	**MS/MS transition (dMRM)**		**Fragementor voltage (V)**	**Collision energy (V)**
		**Presursor ion (*m/z*)**	**Production (*m/z*) (quantifier/qualifier)**		
3-hydroxytyrosol	4.95	153.1	122.4/123.1	95	23/15
isochlorogenic acid	5.14	353.1	191.0/179.0	105	16/16
3,4-dihydroxybenzoic acid	5.34	153.0	109.0/108.1	86	12/28
4-methyl gallic acid	5.67	183.0	168.0/124.1	90	8/16
Catechin	5.94	289.1	245.2/123.1	120	12/36
procyanidin B2	5.97	579.2	127.0/287.1	120	33/37
Epicatechin	6.25	289.1	245.2/203.1	134	12/20
3-hydroxybenzoic acid	6.40	137.0	93.1/N.D.	88	8/N.D.^a^
caffeic acid	6.56	179.0	135.1/89.1	88	16/36
4-hydroxybenzoic acid	6.64	137.0	93.1/65.2	76	16/36
vanillic acid	6.66	167.0	152.0/108.1	80	12/16
Dihydromyricetin	6.75	319.0	193.0/301.0	100	4/8
syringic acid	6.78	197.0	182.0/167.0	90	13/17
resveratrol-3-glycoside	7.03	389.1	227.0/185.0	140	13/37
dihydroferulic acid	7.26	195.1	136.1/121.1	100	11/27
sinapic acid	7.73	223.1	208.0/193.0	90	9/21
Taxifolin	7.73	303.0	285.0/177.0	110	9/9
ferulic acid	7.75	193.1	134.1/N.D.	88	16/N.D.
3-hydroxycinnamic acid	7.82	163.0	119.1/91.1	94	12/28
phenylacetic acid	8.13	135.0	91.2/N.D.	50	4/N.D.
*trans*-2-hydroxycinnamic acid	8.18	163.0	119.1/117.0	80	12/36

*N.D^a^, not detected*.

All calibration curves based on 8–15 points and the calibration curve parameters, coefficient of determination (*r*^2^), linear range, lower limit of detection (LLOD), and lower limit of quantification (LLOQ) of all target analytes are shown in Supplementary Table S2.

### QC of GSPE, RSV Capsules, and CGJ

#### Determination of Weight Uniformity of RSV and GSE Capsules

The mass uniformity of the individual unit dosages contained in each RSV and GSE capsule was performed according to USP, 2091 (19). The calibration of the balance was confirmed prior to the start of the study and at the conclusion of the study. Briefly, 20 intact capsules of each kind of DSs were individually weighed using an electronic balance, and the mass of each capsule content was recorded to 1/10 of a milligram (0.1 mg). After that, the average mass and its SD were calculated. Moreover, the requirements are met if the individual weights lie within the range of 90.0–110.0% of the average weight, and the relative SD (RSD) is ≤ 6.0%.

For any capsules falling within the aforementioned limits, the contents of each capsule should be removed and the emptied shells need to be weighed individually. The net weight could be calculated by subtracting the weight of the shell from the respective gross weight, and the average net content could be determined from the sum of the individual net weights. After that, the difference between each individual net content and the average net content should be determined. The requirements are met if no more than two differences are >10% of the average net content, and in any case the difference does not exceed 25%.

#### Determination of Content Uniformity of the GSPE Capsule

For the preparation of GSE capsules, nine replicates were made in parallel for the QC process. The contents of each capsule were removed with the aid of a small brush or pledget of cotton and dissolved in 50 ml 70% MeOH with 1% FA, vigorously vortexed, and sonicated for 10 min. An aliquot of 100 μl of the extract was diluted by mixing with 0.9 ml 70% MeOH with 1% FA centrifuged at 16,000 rpm for 10 min, and then the supernatant was injected for the LC-UV/Vis-MS analysis.

For reference solutions of RSV capsules, *ca*. 10 mg of *trans*-RSV standard was accurately weighed and diluted to 10 ml using 70% MeOH with 1% FA. The standard stock solution was then sonicated for 10 min and was allowed to cool down to room temperature. An aliquot of 100 μl stock solution was spiked into 0.9 ml 70% MeOH with 1% FA to make the first working solution. Further serial dilutions up to 100~0.1 μg/ml were made using the same solvent. The LC-MS conditions might be the same as mentioned in section QC of Original GSPE Materials.

#### Determination of RSV Capsule Purity and Content Uniformity

For the preparation of RSV capsule samples, nine replicates were made in parallel for the QC process. The contents of each capsule were removed with the aid of a small brush or pledget of cotton and dissolved in 50 ml 70% MeOH with 1% FA. The solution was vigorously vortexed and sonicated for 10 min. An aliquot of 100 μl of the extract was diluted by mixing with 0.9 ml 1% FA in 70% MeOH solution. The solution was centrifuged at 16,000 rpm for 10 min, and then the supernatant was injected for the LC-UV/Vis-MS analysis. The reference standard solution preparation and LC-MS conditions might be the same as described in section QC of Original RSV Materials.

#### Determination of Content Uniformity of CGJ

##### Determination of Anthocyanidins and Flavonols Using LC-UV/Vis-MS

For each of the samples, nine replicates were made in parallel for the QC process. All samples were analyzed without dilution and centrifugated at 12,000 rpm for 10 min. The supernatant was directly injected into UHPLC. All other experimental conditions might be the same as described in section Determination of Anthocyanidins and Flavonols Using LC-UV/Vis-MS.

##### Determination of Other Phenolic Compounds Using UHPLC-QQQ/MS

For the preparation of samples, nine replicates were made in parallel for the QC process. All samples were diluted using 1% FA acidified 70% MeOH solution (1:10). The prepared samples were then centrifugated at 16,000 rpm for 10 min, and the supernatant was directly injected into UHPLC. The reference standard solution preparation and LC-MS conditions might be the same as described in section Determination of Other Phenolic Compounds Using UHPLC-QQQ/MS.

##### Preliminary Stability Study of CGJ

Concord Grape Juice and CGJ placebo bottles were stored in a refrigerator cooler, Heat Craft Unit—Compressor Model #CDT-501H2, Model #CHL 450, with the average storage temperature maintained between 0.6 and 2.22°C at the FDA-approved Rutgers Food Innovation Center, Bridgeton, NJ, USA. From the 36 bottles of CGJ, 3 CGJ samples were randomly selected at each time point as shown in Supplementary Table S10, and for each of the samples, three replicates were made in parallel for the QC process. About 5 ml of CGJ sample was mixed with 5 ml MeOH, vortex for 10 s. Aliquots of 1 ml were transferred to Eppendorf tubes, stored in a paper box, and put it into a −20-degree freezer. While this study was originally designed as a longer-term stability study, for this work described here all the samples were analyzed at the month 6 time point as described in section CGJ Preliminary Stability Study at Month 6 Time Point.

### Dissolution Study of GSPE and RSV Capsules

Grape seed extract and RSV capsules were tested for dissolution based on the recommendations of the FDA and USP 39 general chapters <2040> and <711> (16, 17). Briefly, the two dissolution media, 0.1 N hydrochloric acid (pH 1.2) and 0.05 M acetate buffer (pH 4.6), were evaluated with USP Apparatus 2, 100 rpm rotation speed, and 900 ml dissolution medium. Dissolution profiles were generated over 120 min. Gallic acid, catechin, procyanidin B2, and epicatechin were the marker compounds for GSE capsules, and *trans*-RSV was a marker compound for RSV capsules. Each of these marker compounds was quantified using UHPLC-QQQ/MS. A detailed experiment and the results will be described in a separate report (18).

### Statistical Analysis

Raw UV and MS data were processed using MassHunter Workstation software Data Acquisition (version B.08.00) and Quantitative Analysis (version B.07.01). Data analysis and the production of graphs were performed using R software (version 4.0.5), R studio (version 1.3.959), and Microsoft® Excel for Mac (version 16.49).

## Results and Discussion

### Weight Uniformity Test

The primary purpose of the USP is to provide guidelines for pharmaceutical dosage forms through a series of QC tests, such as identification, dissolution, uniformity of dosage units, assay, moisture, and heavy metal determinations to confirm the products' identity, content, and purity as well as various other chemical, physical, and biological properties. The term “uniformity of dosage unit” is defined as the degree of uniformity in the amount of the drug substance among dosage units (19). Mass uniformity results are presented in Supplementary Table S3, and the GSE capsule weight ranges from 593.70 to 607.90 mg, and for the RSV capsule, the weight range is 551.20–637.20 mg. From the results, all of the observed GSE and RSV capsules within the range of 90.0–110.0% of the average weight had an RSD of 0.56 and 5.30%, respectively, which satisfies the guidelines of the USP <2091> (19).

### RSV Purity and Content Uniformity

#### Method Validation

Numerous studies have reported interesting properties of RSV such as the prevention and treatment of diabetes, cancer, obesity, pain, inflammation, tissue damage, and even aging (7, 12, 20, 21). However, during storage, photochemical and photocatalytic degradation of *trans*-RSV become a problem largely due to the *cis*-isomerization, which occurs when the *trans*-isomer is exposed to sunlight or to artificial or natural UV radiation at the wavelengths of 254 or 366 nm (22–26). Moreover, RSV exists naturally as both *cis-* and *trans*-isomers in nature foods and plants (26). Many reports and data indicate that *cis-* and *trans*-RSV may have different biological effects (21, 27–29). Therefore, we contend that it is necessary to determine the presence and/or absence of *cis*-isomers in RSV capsules. Although HPLC is a popular method to identify and quantify pure compounds, due to the difficulties of isomer separation on the HPLC column, conventional HPLC-UV/Vis method is often not adequate. Thus, a preliminary study was performed to validate the HPLC-UV/Vis method and to prove the *cis-* and *trans-*isomers peaks that were not overlapped and could be clearly separated.

**Figure 2:**
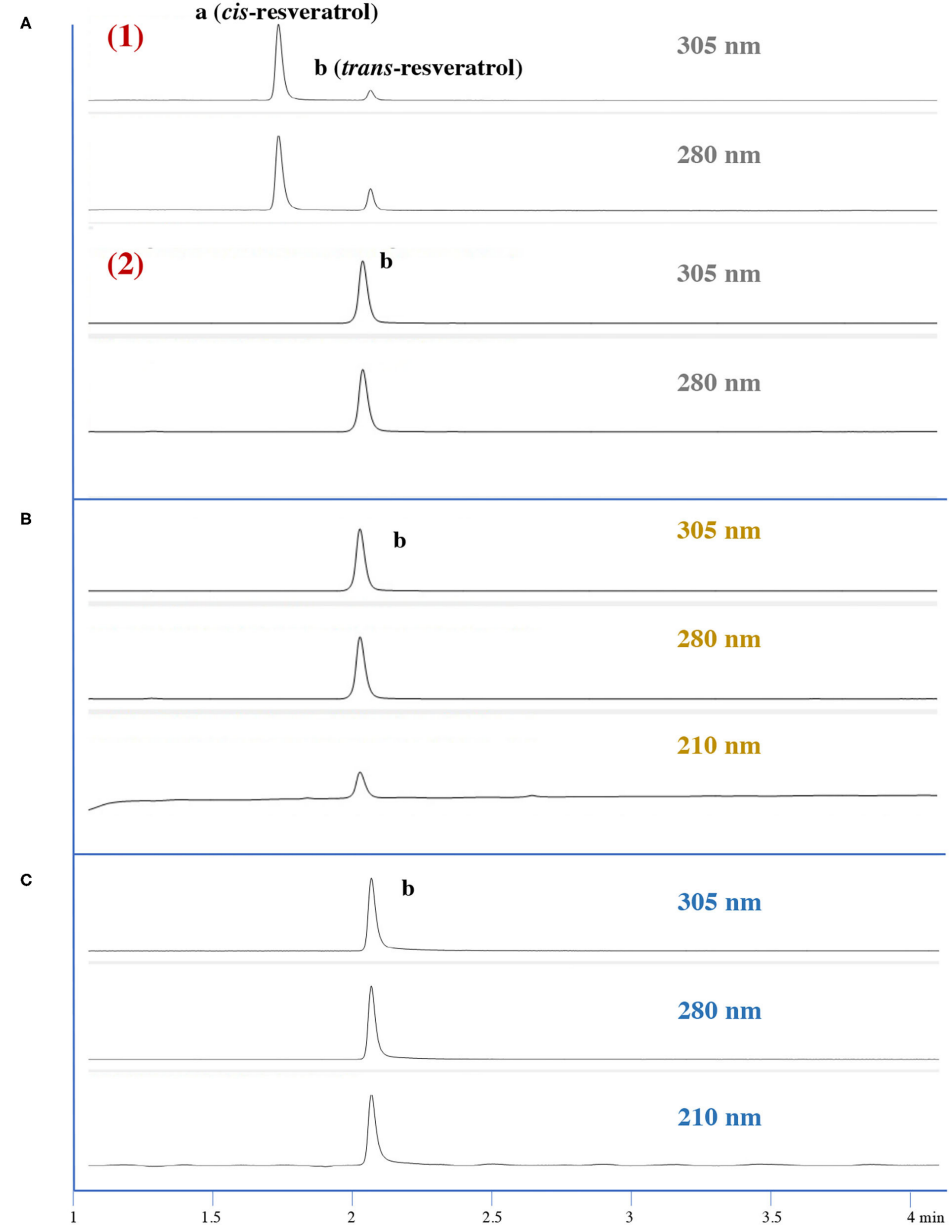
UV chromatographs of **(A)** *trans-* and *cis-*resveratrol (RSV); **(B)** RSV capsules. Peak a, *cis-*RSV, Peak b, *trans*-RSV. **(A)**-(1) UV chromatographs of solution A as described in section RSV Purity and Content Uniformity: Method Validation under 305 and 280 nm; **(A)**-(2) UV chromatographs of solution B under 305 and 280 nm; **(B)** UV chromatographs of the RSV original material under 305, 280, and 210 nm; and **(C)** UV chromatographs of the RSV capsule under 305, 280, and 210 nm.

To prepare *cis*-RSV, the photochemical degradation experiment was performed in our lab based on previous studies (26, 30). Briefly, 2 ml of *ca*. 1 mg/ml *trans*-RSV standard stock solution was stored in a colorless glass vial. The vial was then kept under the sun for the whole day because *trans*-RSV is more easily degraded when irradiated using the entire spectral range rather than using UV and near-UV to visible light (30). The solution from the glass vial (solution A) and the freshly prepared *trans*-RSV standard solution (solution B) both were injected into the HPLC separately, and the chromatographs are presented in Figure 2A. As shown in Figure 2, two major peaks appeared in solution A under 280 and 305 nm, while only one peak (peak b) appeared in solution B, which was the *trans*-RSV peak. The second peak in solution A shares the same retention time and same MS, suggesting that it was also *trans*-RSV. The first peak (peak a) has similar MS, and has the optimum absorbencies at 286 nm, indicating that the new peak is *cis*-RSV. Therefore, this preliminary experiment indicated that our HPLC-UV/Vis is able to separate *trans-* and *cis-*RSV.

#### Purity and Content Uniformity of RSV Original Materials and Capsules

Using our rapid and validated HPLC-UV/V method, our analysis shows that all RSV original materials and capsules were free of *cis*-RSV as well as other impurities and the chromatographs (see Figures 2B,C). The *trans*-RSV content in the RSV capsule is between 97.77 and 102.83% of the average, with a RSD of 1.50%. While we analyzed with the three dosages of 150, 300, and 450 mg; we present only the data with the highest dosage of 450 mg as similar results were obtained for the other two lower dosages. The average content of trans-RSV in RSV capsules was 453.71 mg, which is 0.8% excess of the labeled content (450 mg). The data are presented in detail in Supplementary Table S4.

### GSPE Content Uniformity

Grape seed is a byproduct in the winery and grape juice industry, and contains lipids, proteins, carbohydrates, and 5–8% polyphenols (31). Phenolic compounds in grapes and grape-derived products can be divided into two groups: (a) phenolic acids and related compounds and (b) flavonoids. The most abundant phenolic substances in grape seeds are catechins (catechins, epicatechin, and proanthocyanidins) and their polymers (32). The antioxidant capacities of grape seed proanthocyanidins and natural secondary products have been exhaustively studied. Several reports indicated that grape seed proanthocyanidins have a wide array of positive health effects, including antioxidant, antimicrobial, antiobesity, antidiabetic, anti-neurodegenerative, anti-osteoarthritis, anticancer, and cardio- and eye-protective properties (33). For this reason, almost all GSE DSs on the market claim to have a specific “dosage” of proanthocyanidins. Hence, in this study, the total polyphenol content in the GSPE capsule was first tested, and then a HPLC-UV/Vis-MS method was developed and used to tentatively identify and quantify proanthocyanidin compounds in GSPE capsules.

#### Extracting Solvent Optimization

Phenolic compounds, including polyphenols and proanthocyanidins, vary between the extracts obtained by different solvents. Therefore, a pre-experiment was first performed to compare the extraction efficiency of water and MeOH. Briefly, the content of one GSE capsule was dissolved with 1 L 70% MeOH acidified by 1% FA, and 1 L water acidified by 1% FA. The solvent was sonicated for 30 min. An aliquot of 1,000 μl of the extraction solvent was then centrifugated at 16,000 rpm for 10 min, and the supernatant was directly injected into HPLC. Three replicates were made in parallel for obtaining more accurate results. Gallic acid, epicatechin, procyanidin B2, and epicatechin were quantified using our abovementioned method, and the results are shown in Supplementary Figure S1. For all the four marker compounds, 70% MeOH with 1% FA was more efficient than water with 1% FA. This might be possible because the solubility of those phenolic compounds in water is fairly low, and more water-soluble polysaccharides or other components are extracted as well (34). Hence, 70% MeOH acidified with 1% FA was used to prepare all grape DS products (including RSV and GSE capsules and CGJs) as well as the reference solution. Using this specific solvent to prepare and dilute the standard solution, a high coefficient of determination (*r*^2^) for all the standards was achieved.

#### Content Uniformity and Proanthocyanidin Content in GSPE Original Materials and Capsules

**Figure 3:**
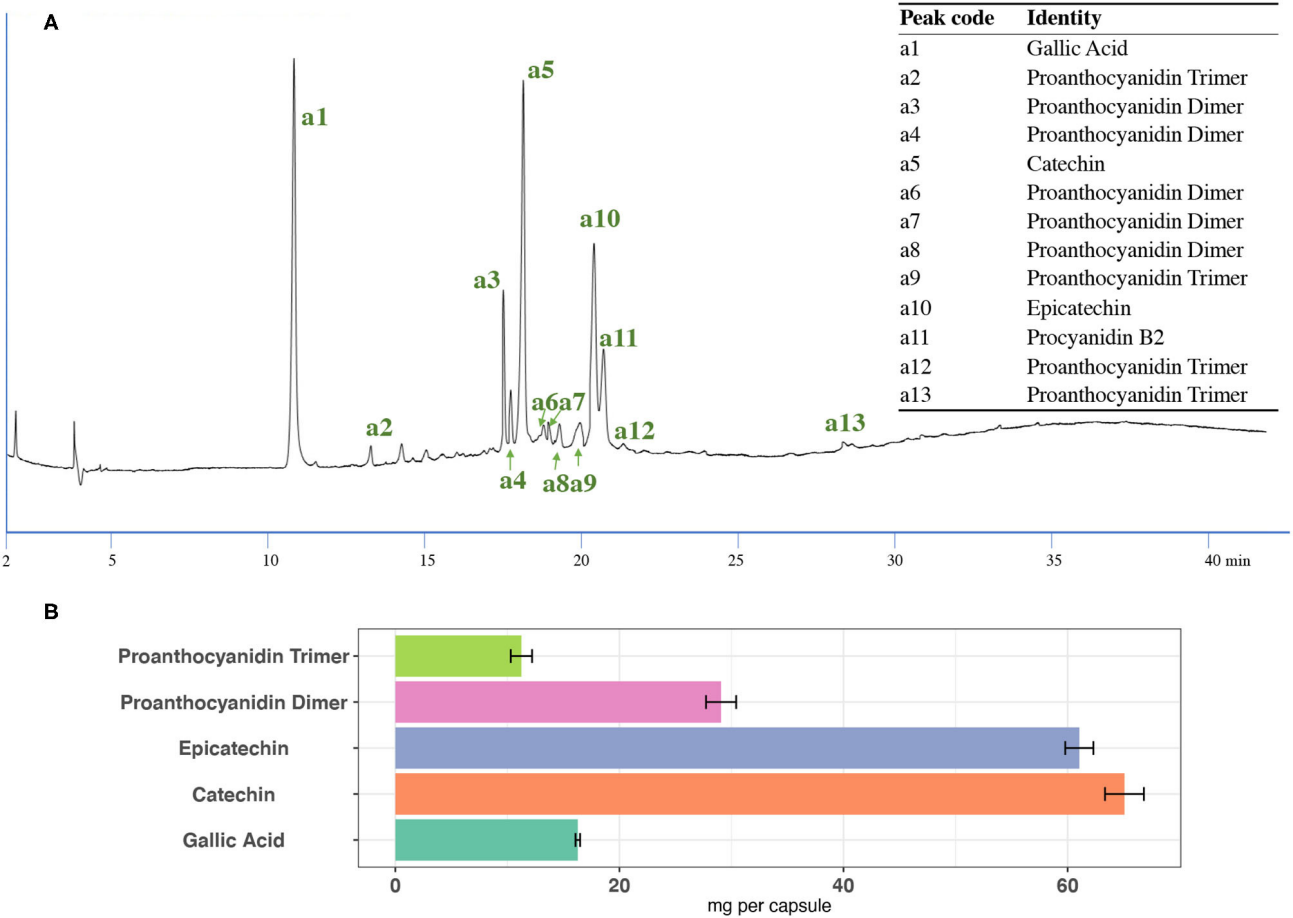
**(A)** UV chromatographs of the Grape Seed Extract (GSE) capsule under 280 nm and the tentative identifies and **(B)** the content of proanthocyanidin trimer, proanthocyanidin dimer, epicatechin, catechin, and gallic acid in the GSE capsule using the optimized extraction method.

The major polyphenols in the GSPE include gallic acid and proanthocyanidins, with monomers of catechin and epicatechin and oligomers, were detected under UV 280 nm. The tentative identification of proanthocyanidin compounds in GSPE was done based on the MS data and published work (6). Figure 3A shows a representative UV chromatogram of the GSPE capsule at 280 nm. The tentative identities and retention times for individual compounds are also listed in Figure 3A. Based on the analysis of MS and UV data and their comparison with the authenticated standards and reported data (6), a total of 13 compounds were simultaneously identified, including gallic acid, catechin, epicatechin, 4 proanthocyanidin trimers, and 6 proanthocyanidin dimers. All tentatively identified proanthocyanidin compound values in the GSPE are reported in Supplementary Table S5 and illustrated in Figure 3B.

For the GSPE original material, the results show that the total identified polyphenols together with gallic acid content in the five subsamples are very similar, with a mean value of 33.37% and a RSD of 3.24%. These results suggest that the GSPE original material was well-homogenous. For the GSPE capsules, the results show that the content of total identified proanthocyanidin compounds together with gallic acid in each capsule is also very close, with a mean value of 182.75 ± 4.07 mg and an RSD of 1.43%. Moreover, catechin and epicatechin are the major proanthocyanidin compounds in the GSPE capsules. Each capsule contains 65.07 ± 2.01 mg catechin and 61.05 ± 1.76 mg epicatechin, as well as 166.45 ± 3.70 mg of all tentatively identified proanthocyanidin compounds, which ensured the daily taking of 95 mg of proanthocyanidins (35). All these results suggest that GSPE capsules show high quality and homogeneity.

### CGJ Content Uniformity

Concord Grape Juice contains a variety of phenolic compounds, including anthocyanins and proanthocyanidins and relatively high levels of total phenolics (10). Anthocyanins include red, blue, or purple plant pigments (9). Many *in vitro* and *in vivo* studies have indicated that grape anthocyanins appear to exert health benefit effects, including the prevention of various diseases, such as neuronal and cardiovascular illnesses, cancer, and diabetes, in which reactive radical species are integral to disease development and progression (4–6). Therefore, the total polyphenol content in CGJ was tested, a HPLC-UV/Vis method was used to tentatively identify and quantify the anthocyanidin and flavanol, and a UHPLC-QQQ/MS methodology was used to quantify phenolic compounds in CGJ.

#### Content of Anthocyanins, Flavonols in CGJ

**Figure 4:**
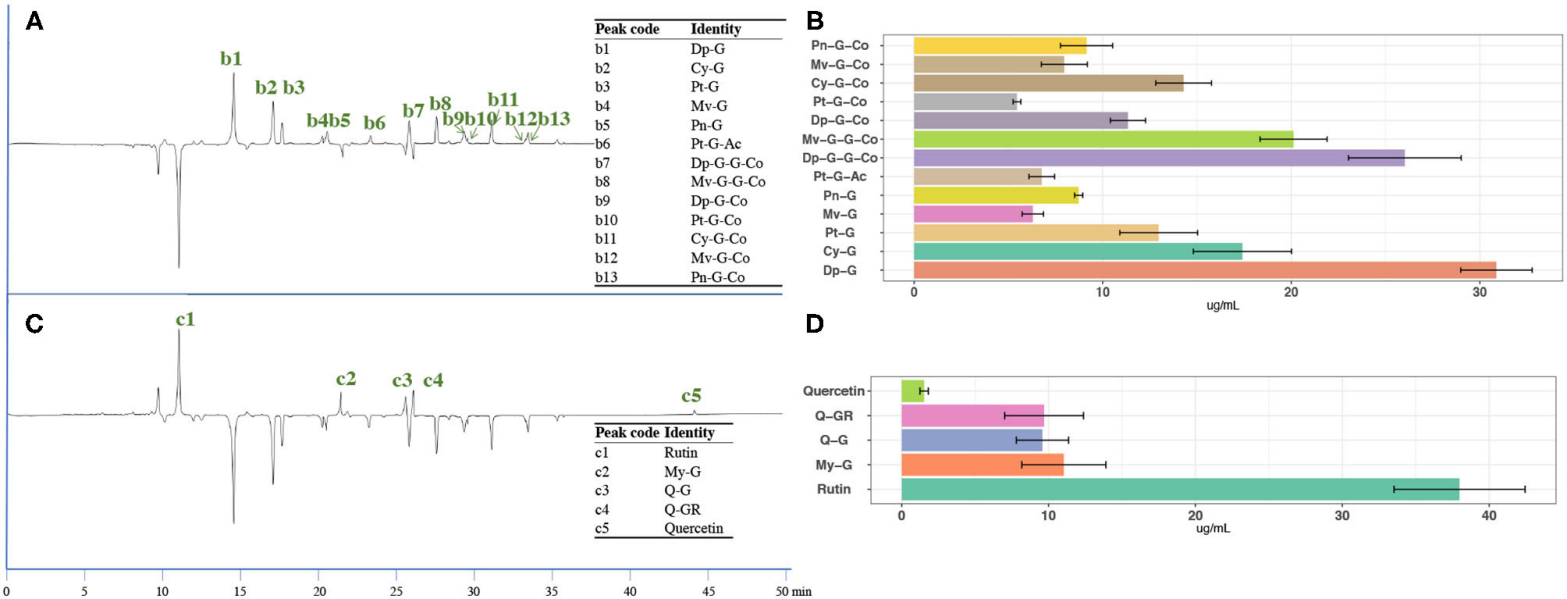
**(A)** UV chromatographs of CGJ under 520 nm and the tentative identification; **(B)** the content of detected anthocyanins in CGJ; **(C)** UV chromatographs of CGJ under 370 nm and the tentative identification; and **(D)** the content of the detected flavonols in CGJ. Dp, delphinidin; Cy, cyanidin; Pt, petunidin; Mv, malvidin; Pn, peonidin; Pt, petunidin; My, myricetin; Q, quercetin; G, glucoside or other hexoside; Ac, acetyl; Co, coumaroyl; GR, glucuronoyl.

The representative UV chromatograms at 520 nm (for anthocyanins) and 370 nm (for flavonols) of CGJ are illustrated in Figures 4A,C. The identification is done based on the UV data and published report (6), and a total of 13 anthocyanins (delphinidin glucoside, cyanidin glucoside, petunidin glucoside, malvidin glucoside, peonidin glucoside, petunidin acetyl glucoside, delphinidin coumaroyl diglucoside, malvidin coumaroyl diglucoside, delphinidin coumaroyl glucoside, petunidin coumaroyl glucoside, cyanidin coumaroyl glucoside, malvidin coumaroyl glucoside, and peonidin coumaroyl glucoside), and 5 flavonols (rutin, myricetin glucoside, quercetin glucuronoyl, and quercetin) were tentatively identified. The content of all identified anthocyanins and flavonols in CGJ samples is presented in Figures 4B,D, and the details of data are provided in Supplementary Tables S7, S8. From these results, the total concentration of the identified anthocyanins was 177.39 ± 6.67 μg/ml with an RSD of 3.91%, for the flavonols, the value was 69.82 ± 4.66 μg/ml with an RSD of 5.49%. These data suggest that CGJ is a rich source of grape anthocyanins and flavonols, and that CGJ is well-homogeneous. Among all the identified compounds, rutin is the most abundant one in CGJ, with a content of 32.34–43.90 μg/ml. Rutin is a common dietary flavonoid and has been reported to possess diverse pharmacological activities, including antioxidant, anti-inflammatory, anticancer, antidiabetic, antimicrobial, and neuroprotection effects (36). However, due to the low aqueous solubility, poor stability and limited membrane permeability, bioavailability of rutin is very poor, and the observed effects *in vitro* do not always translate into clinical outcomes (36, 37). Hence, those observations and connections indicate a need to improve CGJ DSs to enhance the bioavailability of rutin and other flavonoids.

#### Content of Other Phenolic Compounds in CGJ

Even though HPLC-UV/Vis is generally used for the identification and quantification of phenolic compounds from grapes and their products, some compounds are difficult to be effectively separated and accurately identified using the methodology due to their insufficient peak capacity and the accumulation of analytes (38). UHPLC coupled with triple quadrupole MS (UHPLC-QQQ-MS/MS) based on HPLC using a small particle diameter column and mass spectrometer allows rapid screening of a large number of phytochemicals using the information on the characteristics of molecular ions (38, 39). Multiple-reaction monitoring (MRM) mode of QQQ-MS/MS is a highly specific and sensitive MS technique that can selectively quantify the compounds within complex mixtures. It selects specific analytes and absolute quantitation of proteins, peptides, metabolites, and lipids in the fields of biochemistry, drug metabolism, and plant studies (39, 40, 44). In contrast to the UV chromatograms of GSE capsules, the CGJs are more complicated. In part, this may be due to the loss of some phenolic compounds because of the solubility during the extraction process, while the removal of extraction solvents may meanwhile destroy the thermal-labile compounds. Taking into account that the sensitivity of this methodology is higher than that of the UV/Vis detector, and the complexity of CGJ, the MRM mode of UPLC-QQQ-MS/MS was next used to analyze all CGJ samples.

**Figure 5:**
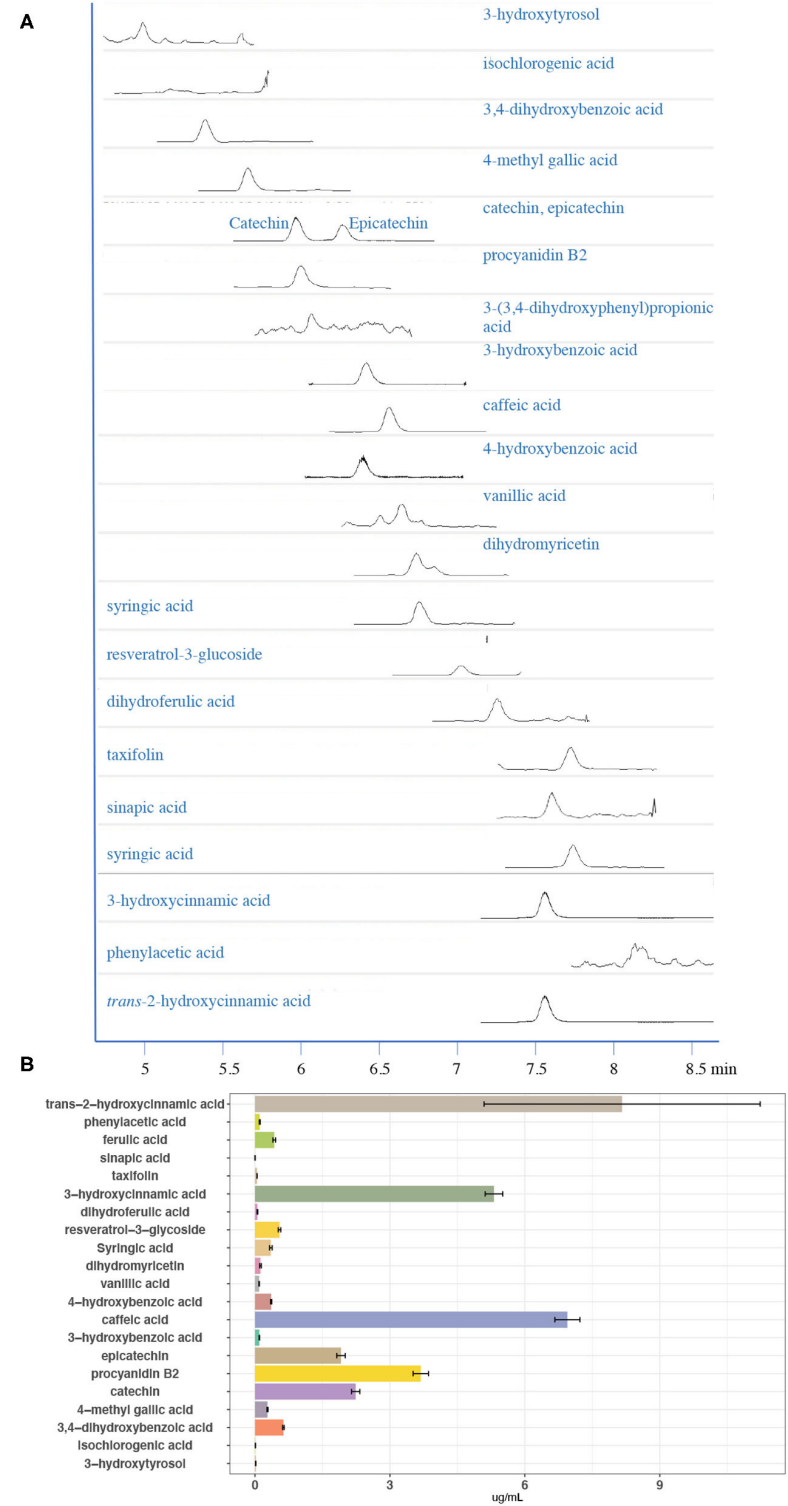
**(A)** Mass spectrometry (MS) chromatographs of CGJ and **(B)** the content of targeted compounds in CGJ.

Due to highly priced commercial phenolic compound standards, we first screened the CGJ using our published UHPLC-QQQ-MS/MS method (41), and identified 21 phenolic compounds in CGJ DS samples, including 3-hydroxytyrosol isochlorogenic acid, 3,4-dihydroxybenzoic acid, 4-methyl gallic acid, catechin, procyanidin B2, epicatechin, 3-hydroxybenzoic acid, caffeic acid, 4-hydroxybenzoic acid, vanillic acid, dihydromyricetin, syringic acid, resveratrol-3-glycoside, dihydroferulic acid, 3-hydroxycinnamic acid, taxifolin, sinapic acid, ferulic acid, phenylacetic acid, and *trans*-2-hydroxycinnamic acid. Then, the UHPLC-QQQ-MS/MS method was optimized based on 21 phenolic compounds. A well-separated peak for each compound standard was achieved as shown in Figure 5A. Standard curve linearity, detection limits, precision, and recovery of phenolic compounds are shown in Supplementary Table S1.

Determination of the contents of each of the targeted phenolic compounds in the CGJ and CGJ concentrate and the results are presented in Supplementary Table S9, Figure 5B. Among the 21 phenolic compounds, the most abundant compound in CGJ is *trans*-2-hydroxycinnamic acid, with a concentration of 10.46 ± 0.50 μg/ml. Moreover, the CGJ DS also has a high concentration of caffeic acid and 3-hydroxycinnamic acid, with the values of 8.90 ± 0.24 and 7.72 ± 0.25 μg/ml. All three compounds belong to the hydroxycinnamic acid, which is an important class of polyphenolic compounds originated from the Mevolanate-Shikimate biosynthesis pathways in plants and possess potent antioxidant and anti-inflammatory properties (42). Recent publications and data have confirmed the important role of those kinds of hydroxycinnamic acid class compounds in the prevention and treatment of obesity, diabetes, and associated disorders (43). The total content of all the identified phenolic compounds using our optimized UHPLC-QQQ-MS/MS was 42.21 ± 1.28 μg/ml with an RSD of 2.10%. Overall, our results clearly revealed that the CGJ DS is a rich source of phenolic compounds and CGJ is well-homogeneous, suggesting that the manufacturing system meets the USP standardization.

#### CGJ Preliminary Stability Study at Month 6 Time Point

While this part of experiment with CGJ is still in progress, the data in detail are presented in Supplementary Table S11. For CGJ, we found that under proper cold storage conditions (4°C), with the bottled product kept in the packaged and shipping cartons and in absence resulted in a stable shelf-life for anthocyanidins, flavanols, and phenols up to month 6, at which time these compounds decreased to 85.70, 95.52, and 94.98%, respectively, of their original contents. The total concentration of all the identified compounds also decreased to 89.20%. Taking into account that anthocyanidins are more unstable than flavanols and phenols, the degradation rate of anthocyanidins was higher as expected than the others. More research is needed to fully understand the degradation profile of CGJ and to identify the strategies to further extend its shelf-life in storage. Our data show that under our experimental conditions the CGJ DS is stable until month 6 after which a new batch with the same needed chemical profile should the clinical trial continue beyond that time point.

## Conclusion

In the present study, we extensively analyzed three *V. vinifera-* (grape-) based DSs, including GSE and RSV capsules, and CGJ, using our optimized LC-UV/Vis-MS and UHPLC-QQQ/MS methods. The weight variation of GSE and RSV capsules was also evaluated according to the USP tests. The total polyphenol content in the three products was also tested. From the results, all RSV and GSE capsules satisfy or meet the guidelines of the USP <2091> (19). Moreover, GSE capsules and CGJ both possessed a high polyphenol content according to the total polyphenol content test. All RSV capsules were free of *cis*-RSV as well as other impurities from our optimized HPLC-UV/Vis-MS, and the average content of *trans*-RSV in RSV capsules only exceeded the labeled content by 0.8%. Meanwhile, the chemical fingerprinting using the HPLC-UV/Vis-MS method displayed that the content of total identified proanthocyanidin compounds together with gallic acid in each GSE capsule is very similar, and the GSE capsule is a good resource of catechin and epicatechin, with 65.07 ± 2.01 mg and 61.05 ± 1.76 mg per capsule, respectively. Thirteen anthocyanins and five flavonols were identified and quantified using the HPLC-UV/Vis-MS methodology, with the total concentration of the identified anthocyanins and flavonols being 177.39 ± 6.67 μg/ml and 69.82 ± 4.66 μg/ml. Finally, the optimized UHPLC-QQQ/MS method was used to quantify 21 phenolic compounds in CGJ, and this DS showed a high concentration of *trans*-2-hydroxycinnamic acid, caffeic acid, and 3-hydroxycinnamic acid. The present study provides a comprehensive overall QC for grape-derived DSs, and the results show that a careful strategic approach to the authentication of each botanical ingredient to be used in clinical trials needs to follow the NIH guidelines on natural product integrity to avoid the issues of adulteration (13). Given the complexity of these and most botanical products from the issues of purity, quality, adulteration, consistency, and coupled to the complex chemistry found in grape-derived botanicals, such an approach is required to ensure that each of the materials used is homogeneous and stable and contain specific concentrations and profiles of bioactives to provide the needed solid foundation upon which clinical trials are conducted with the goal of realizing measurable mental health outcomes such as reducing depression and anxiety and understanding of their underlying biological mechanisms.

## Data Availability Statement

The original contributions presented in the study are included in the article/Supplementary Material, further inquiries can be directed to the corresponding author/s.

## Author Contributions

WL, JS, and QW designed the experiment. WL executed the experiment, performed the data analysis, and drafted the manuscript. JS and QW supervised the overarching project. WL, DR, MF, GP, and JM discussed the original concept and overall objectives. DR and MF provided key insights into botanical ingredients and formulation. GP and JM provided criteria under which the botanicals were needed for clinical trial applications. All authors contributed to reviewing, editing, and strengthening of the manuscript.

## Funding

This study was supported by grant U19 AT010835 from the Office of DSs (ODS), the National Center for Complementary and Integrative Health (NCCIH) and the National Institute on Aging (NIA) of the NIH in support of *Influence of Dietary Botanical Supplements on Biological and Behavioral Resilience* awarded to the Icahn School of Medicine at Mount Sinai. GP holds a Senior Scientist Award. We acknowledge that the contents of this study do not represent the views of the NCCIH or the US Government, or the US Department of Veterans Affairs. Partial support also was provided by the New Jersey Agriculture Experiment Station, HATCH project NJ12170.

## Conflict of Interest

DR was employed by company Eagle Nutritionals. The remaining authors declare that the research was conducted in the absence of any commercial or financial relationships that could be construed as a potential conflict of interest.

## Publisher's Note

All claims expressed in this article are solely those of the authors and do not necessarily represent those of their affiliated organizations, or those of the publisher, the editors and the reviewers. Any product that may be evaluated in this article, or claim that may be made by its manufacturer, is not guaranteed or endorsed by the publisher.
