# Supplementary material for: Chemical, Manufacturing, and Standardization Controls of Grape Polyphenol Dietary Supplements in Support of a Clinical Study: Mass Uniformity, Polyphenol Dosage, and Profiles
Source: Front Nutr. 2021 Dec 16;8:780226. doi: 10.3389/fnut.2021.780226 (PMC8716858; doi:10.3389/fnut.2021.780226)
Supplement: Supplementary file 1 [file Table_1.docx]

**Chemistry, Manufacturing and Standardization Controls of Grape Polyphenol** **Dietary Supplements for Support of a Clinical Study: Mass Uniformity, Polyphenol Dosage and Profile**

**Weiting Lyu ^a, b^, David Rodriguez ^c^, Mario G. Ferruzzi ^d^, Giulio M. Pasinetti ^e, f^, James W. Murrough ^e, g^, James E. Simon ^a, b, *^ and Qingli Wu ^a, b, *^**

^a^ New Use Agriculture and Natural Plant Products Program, Department of Plant Biology and Center for Agricultural Food Ecosystems, Institute of Food, Nutrition & Health, Rutgers University, 59 Dudley Road, New Brunswick, NJ 08901, USA

^b^ Department of Medicinal Chemistry, Ernest Mario School of Pharmacy, Rutgers University, 160 Frelinghuysen Road, Piscataway, NJ 08854, USA

^c^ Eagle Nutritionals, 485 Washington Ave, Carlstadt, NJ 07072, USA

^d^ Department of Food, Bioprocessing and Nutrition Sciences, Plants for Human Health Institute, North Carolina State University, 600 Laureate Way, Kannapolis, NC 28081, USA

^e^ Department of Neurology, Icahn School of Medicine at Mount Sinai, 1 Gustave L. Levy Place, New York, NY 10029, USA

^f^ Geriatric Research, Education and Clinical Center, James J. Peters Veterans Affairs Medical Center, Bronx, NY, USA

^g^ Depression and Anxiety Center for Discovery and Treatment, Department of Psychiatry, Icahn School of Medicine at Mount Sinai, 1 Gustave L. Levy Place, New York, NY 10029, USA

**^*^** **Correspondence**

James E. Simon and Qingli Wu

E-mail addresses: jimsimon@rutgers.edu (J.E. Simon), qlwu@sebs.rutgers.edu (Q. Wu).

**Table S1.** Calibration curve parameters of Sections 2.4.1 and 2.5.2, Sections 2.5.3, Sections 2.4.3.1 and 2.5.4.1.

|  | **Compound** | **Liner range (μg/mL )** | **LLOD^b^** | **LLOQ^c^** | **equation** | **r^2 a^** |
| --- | --- | --- | --- | --- | --- | --- |
| Section 2.4.1 & 2.5.2 | gallic acid | 0.19~50 | 0.095 | 0.190 | y = 4.7376x - 1.6694 | 0.9985 |
|  | catechin | 0.165~42.307 | 0.083 | 0.165 | y = 0.9669x + 0.5853 | 0.9909 |
|  | epicatechin | 0.180~46.152 | 0.090 | 0.180 | y = 1.636x - 0.7367 | 0.9967 |
|  | procyanidin B2 | 0.185~47.3 | 0.093 | 0.185 | y = 0.8876x + 0.046 | 0.9958 |
|  | procyanidin C1 | 0.155~39.584 | 0.078 | 0.155 | y = 1.2575x + 0.9748 | 0.994 |
| Section 2.5.3 | *trans*-resveratrol | 0.215~110.200 | 0.108 | 0.215 | y = 24.883x + 19.051 | 0.9969 |
| Section 2.4.3.1 & 2.5.4.1 | cyanidin-3-glucoside | 2.026~1037.067 (μmol/L) | 1.103 | 2.206 | y = 3.5774x - 23.287 | 0.9988 |
|  | quercetin | 1.470~752.723 (μmol/L) | 0.735 | 1.470 | y = 2.3249x + 10.403 | 0.9979 |

r^2 a^, the coefficient of determination; LLOD^b^, lower limit of detection; LLOQ^c^, lower limit of quantitation.

**Table S2.** Calibration curve parameters for Sections 2.4.3.2 and 2.5.3.2.

| **Compound** | **Equation** | **r^2 a^** | **LLOD^b^** | **LLOQ^c^** | **Linear Range** |
| --- | --- | --- | --- | --- | --- |
| 3-hydroxytyrosol | y=3.7046x+1.0160 | 0.9931 | 0.2407 | 0.4904 | 0.49~419.25 |
| isochlorogenic acid | y=84.9248x+12.3897 | 0.9976 | 0.2199 | 0.4399 | 0.44~3603.50 |
| 3,4-dihydroxybenzoic acid | y=62.4158x+42.0946 | 0.9980 | 0.3959 | 0.7919 | 0.79~810.88 |
| 4-methyl gallic acid | y=38.4160x+4.7401 | 0.9959 | 0.2051 | 0.4103 | 0.41~840.25 |
| catechin | y=65.8718x+1402.1584 | 0.9964 | N.D. | 0.2432 | 0.24~995.25 |
| procyanidin B2 | y=8.1154x+7.9475 | 0.9906 | 0.5076 | 1.0151 | 1.02~1039.50 |
| epicatechin | y=47.7214x+855.7486 | 0.9969 | N.D. | 0.2432 | 0.24~995.25 |
| 3-hydroxybenzoic acid | y=94.9302x+326.3861 | 0.9926 | 0.2200 | 0.4399 | 0.44~450.5 |
| caffeic acid | y=80.1515x+30.9059 | 0.9922 | 1.7258 | 3.4517 | 3.45~1767.25 |
| 4-hydroxybenzoic acid | y=30.2633x+158.6020 | 0.9914 | 0.8629 | 1.7258 | 1.73~883.63 |
| vanillic acid | y=5.6089x+2.4118 | 0.9974 | 3.7900 | 7.5801 | 7.58~1940.50 |
| dihydromyricetin | y=110.0414x+48.5365 | 0.9891 | N.D. | 0.2834 | 0.2834~580.44 |
| syringic acid | y=7.2802x+4.7162 | 0.9952 | 0.4695 | 1.8782 | 1.88~1923.50 |
| resveratrol-3-glycoside | y=102.1807x+300.1219 | 0.9918 | N.D. | 0.2432 | 0.24~124.53 |
| dihydroferulic acid | y=10.6654x-0.5518 | 0.9992 | 0.4146 | 1.6582 | 1.66~1698.00 |
| sinapic acid | y=20.8051x+4.9203 | 0.9922 | 0.4483 | 1.4286 | 1.43~918.13 |
| taxifolin | y=194.3957x+81.2112 | 0.9901 | 0.2073 | 0.8291 | 0.83~424.50 |
| ferulic acid | y=11.9829x+1.4259 | 0.9932 | 0.4865 | 1.9458 | 1.95~498.13 |
| 3-hydroxycinnamic acid | y=92.2362x+548.6787 | 0.9993 | 0.2221 | 0.4442 | 0.44~454.81 |
| phenylacetic acid | y=4.0946x-15.0578 | 0.9960 | 0.4442 | 1.7766 | 1.78~3638.5 |
| *trans*-2-hydroxycinnamic acid | y=79.9507x+21.4687 | 0.9980 | 3.3838 | 6.7676 | 6.77~866.25 |

r^2 a^, the coefficient of determination; LLOD^b^, lower limit of detection; LLOQ^c^, lower limit of quantitation.

**Table S3.** Mass uniformity results.

| **RSV capsule** | | **GSE capsule** | |
| --- | --- | --- | --- |
| RSV sample 1 | 637.20 | GSE sample 1 | 606.10 |
| RSV sample 2 | 615.40 | GSE sample 2 | 599.00 |
| RSV sample 3 | 607.70 | GSE sample 3 | 607.90 |
| RSV sample 4 | 551.20 | GSE sample 4 | 605.50 |
| RSV sample 5 | 624.20 | GSE sample 5 | 604.50 |
| RSV sample 6 | 569.20 | GSE sample 6 | 604.00 |
| RSV sample 7 | 633.50 | GSE sample 7 | 601.80 |
| RSV sample 8 | 562.80 | GSE sample 8 | 599.80 |
| RSV sample 9 | 559.10 | GSE sample 9 | 598.50 |
| RSV sample 10 | 617.30 | GSE sample 10 | 604.70 |
| RSV sample 11 | 635.10 | GSE sample 11 | 601.40 |
| RSV sample 12 | 615.10 | GSE sample 12 | 593.70 |
| RSV sample 13 | 554.60 | GSE sample 13 | 605.20 |
| RSV sample 14 | 625.70 | GSE sample 14 | 600.20 |
| RSV sample 15 | 625.40 | GSE sample 15 | 600.70 |
| RSV sample 16 | 561.10 | GSE sample 16 | 601.80 |
| RSV sample 17 | 572.50 | GSE sample 17 | 599.50 |
| RSV sample 18 | 628.00 | GSE sample 18 | 598.70 |
| RSV sample 19 | 620.60 | GSE sample 19 | 601.60 |
| RSV sample 20 | 633.60 | GSE sample 20 | 598.70 |
| mean | **602.47** | mean | **601.67** |
| SD^a^ | 31.94 | SD | 3.39 |
| SD^a^ | 5.30% | RSD | 0.56% |

SD^a^, standard deviation; RSD^b^, relative standard deviation.

**Table S4.** *Trans*-resveratrol content in RSV capsules (label: 450 mg).

| **Sample code** | ***trans*-resveratrol content per capsule** |
| --- | --- |
| RSV sample 1 | 446.27 (98.36%) |
| RSV sample 2 | 466.53 (102.83%) |
| RSV sample 3 | 453.11 (99.87%) |
| RSV sample 4 | 458.32 (101.02%) |
| RSV sample 5 | 447.47 (98.62%) |
| RSV sample 6 | 460.11 (101.41%) |
| RSV sample 7 | 453.07 (99.86%) |
| RSV sample 8 | 443.58 (97.77%) |
| RSV sample 9 | 454.9 (100.26%) |
| mean | **453.71** |
| SD^a^ | 6.88 |
| RSD^b^ | 1.50% |

SD^a^, standard deviation; RSD^b^, relative standard deviation.

**Table** **S5**. Tentatively identified proanthocyanidin compounds.

| **Peak code** | **a1** | **a2** | **a3** | **a4** | **a5** | **a6** | **a7** | **a8** | **a9** | **a10** | **a11** | **a12** | **a13** |
| --- | --- | --- | --- | --- | --- | --- | --- | --- | --- | --- | --- | --- | --- |
| Identity | GA^a^ | P3^b^ | P2^c^ | P2 | C^d^ | P2 | P2 | P2 | P3 | EC^e^ | P2(B2^f^) | P3 | P3 |
| Bottom1 | 2.88% | 0.37% | 1.78% | 0.66% | 11.74% | 0.22% | 0.34% | 0.36% | 1.13% | 11.48% | 2.05% | 0.34% | 0.58% |
| Bottom2 | 3.01% | 0.32% | 1.91% | 0.66% | 12.25% | 0.23% | 0.33% | 0.37% | 1.13% | 11.13% | 1.90% | 0.37% | 0.54% |
| Bottom3 | 3.02% | 0.32% | 1.99% | 0.66% | 11.96% | 0.25% | 0.35% | 0.38% | 1.05% | 11.49% | 1.90% | 0.31% | 0.57% |
| Average | 2.97% | 0.34% | 1.89% | 0.66% | 11.98% | 0.23% | 0.34% | 0.37% | 1.10% | 11.37% | 1.95% | 0.34% | 0.56% |
| Center1 | 2.98% | 0.32% | 1.97% | 0.66% | 11.08% | 0.21% | 0.36% | 0.38% | 1.08% | 11.25% | 1.70% | 0.33% | 0.52% |
| Center2 | 3.11% | 0.33% | 1.94% | 0.65% | 11.31% | 0.21% | 0.33% | 0.40% | 1.19% | 11.60% | 1.60% | 0.33% | 0.48% |
| Center3 | 3.07% | 0.43% | 1.96% | 0.64% | 11.14% | 0.21% | 0.32% | 0.38% | 1.12% | 11.23% | 1.72% | 0.33% | 0.55% |
| Average | 3.06% | 0.36% | 1.96% | 0.65% | 11.18% | 0.21% | 0.34% | 0.39% | 1.13% | 11.36% | 1.68% | 0.33% | 0.52% |
| Left1 | 2.59% | 0.45% | 1.88% | 0.54% | 12.46% | 0.17% | 0.31% | 0.29% | 1.07% | 9.36% | 1.74% | 0.26% | 0.51% |
| Left2 | 2.54% | 0.46% | 1.76% | 0.61% | 13.07% | 0.18% | 0.35% | 0.30% | 1.08% | 10.13% | 1.77% | 0.31% | 0.47% |
| Left3 | 2.60% | 0.49% | 1.61% | 0.53% | 13.21% | 0.20% | 0.30% | 0.29% | 1.10% | 9.94% | 1.78% | 0.29% | 0.49% |
| Average | 2.58% | 0.47% | 1.75% | 0.56% | 12.91% | 0.18% | 0.32% | 0.29% | 1.08% | 9.81% | 1.76% | 0.29% | 0.49% |
| Right1 | 2.94% | 0.51% | 1.63% | 0.69% | 11.11% | 0.23% | 0.33% | 0.37% | 1.15% | 10.55% | 2.20% | 0.34% | 0.54% |
| Right2 | 2.99% | 0.41% | 1.90% | 0.62% | 10.78% | 0.20% | 0.34% | 0.41% | 1.14% | 10.69% | 2.03% | 0.39% | 0.60% |
| Right3 | 2.96% | 0.38% | 1.41% | 0.49% | 10.92% | 0.22% | 0.34% | 0.39% | 1.09% | 10.54% | 2.10% | 0.32% | 0.60% |
| Average | 2.96% | 0.43% | 1.65% | 0.60% | 10.94% | 0.22% | 0.34% | 0.39% | 1.13% | 10.59% | 2.11% | 0.35% | 0.58% |
| Top1 | 3.00% | 0.41% | 1.34% | 0.59% | 13.06% | 0.09% | 0.30% | 0.34% | 1.05% | 11.16% | 1.86% | 0.33% | 0.53% |
| Top2 | 3.01% | 0.40% | 1.24% | 0.67% | 13.62% | 0.08% | 0.28% | 0.34% | 1.05% | 11.59% | 2.05% | 0.35% | 0.55% |
| Top3 | 3.06% | 0.44% | 1.36% | 0.65% | 13.20% | 0.08% | 0.36% | 0.35% | 1.06% | 11.79% | 2.08% | 0.31% | 0.46% |
| Average | 3.02% | 0.42% | 1.32% | 0.64% | 13.29% | 0.09% | 0.32% | 0.34% | 1.06% | 11.51% | 2.00% | 0.33% | 0.51% |

GA^a^, gallic acid; P3^b^, proanthocyanidin trimer; P2^c^, proanthocyanidin dimer; C^d^, catechin; EC^e^, epicatechin; B2^f^, procyanidin B2. All data expressed as rel %’s in the Grape Seed Exrtract (GSE) and by location in the original material.

**Table S6**. Content of tentatively identified proanthocyanidin compounds values in Grape Seed Extracts (GSE) capsules.

| **Peak code** | **a1** | **a2** | **a3** | **a4** | **a5** | **a6** | **a7** | **a8** | **a9** | **a10** | **a11** | **a12** | **a13** |
| --- | --- | --- | --- | --- | --- | --- | --- | --- | --- | --- | --- | --- | --- |
| Identity | GA^a^ | P3^b^ | P2^c^ | P2 | C^d^ | P2 | P2 | P2 | P3 | EC^e^ | P2(B2^f^) | P3 | P3 |
| GSE sample1 | 16.31 | 1.68 | 9.93 | 3.01 | 66.54 | 1.26 | 2.05 | 1.99 | 5.72 | 60.95 | 10.41 | 2.17 | 2.95 |
| GSE sample2 | 16.00 | 1.56 | 9.69 | 3.25 | 66.67 | 1.08 | 1.81 | 1.56 | 5.23 | 61.01 | 10.41 | 1.68 | 2.35 |
| GSE sample3 | 16.25 | 1.56 | 10.11 | 3.07 | 67.09 | 1.08 | 1.44 | 2.11 | 6.14 | 60.53 | 9.99 | 1.26 | 2.59 |
| GSE sample4 | 16.18 | 1.74 | 10.41 | 3.19 | 65.70 | 1.08 | 1.38 | 1.99 | 5.84 | 58.42 | 9.69 | 1.50 | 1.74 |
| GSE sample5 | 16.25 | 1.87 | 10.65 | 3.25 | 66.06 | 1.20 | 1.44 | 2.05 | 6.38 | 60.29 | 10.77 | 1.26 | 2.71 |
| GSE sample6 | 16.37 | 1.87 | 10.77 | 2.95 | 62.51 | 1.14 | 1.38 | 1.99 | 5.11 | 61.73 | 10.65 | 1.20 | 1.08 |
| GSE sample7 | 16.49 | 1.81 | 10.95 | 3.25 | 62.87 | 1.08 | 1.74 | 2.53 | 6.92 | 61.91 | 10.53 | 1.26 | 1.02 |
| GSE sample8 | 16.67 | 1.62 | 12.09 | 3.49 | 64.68 | 1.08 | 1.93 | 2.53 | 6.68 | 61.79 | 10.77 | 1.32 | 2.17 |
| GSE sample9 | 16.06 | 1.81 | 11.01 | 3.37 | 63.54 | 0.96 | 1.74 | 2.17 | 4.93 | 62.81 | 10.23 | 1.26 | 3.25 |

GA^a^, gallic acid; P3^b^, proanthocyanidin trimer; P2^c^, proanthocyanidin dimer; C^d^, catechin; EC^e^, epicatechin; B2^f^, procyanidin B2. All data in mg per capsule.

**Table S7.** Content of tentatively identified anthocyanins in Concord Grape Juice (CGJ).

|  | **b1** | **b2** | **b3** | **b4** | **b5** | **b6** | **b7** | **b8** | **b9** | **b10** | **b11** | **b12** | **b13** |
| --- | --- | --- | --- | --- | --- | --- | --- | --- | --- | --- | --- | --- | --- |
| **Compound** | **Dp-G** | **Cy-G** | **Pt-G** | **Mv-G** | **Pn-G** | **Pt-G-Ac** | **Dp-G-G-Co** | **Mv-G-G-Co** | **Dp-G-Co** | **Pt-G-Co** | **Cy-G-Co** | **Mv-G-Co** | **Pn-G-Co** |
| **RT^a^ (min)** | **14.22** | **16.70** | **17.25** | **19.79** | **20.09** | **22.82** | **25.25** | **26.96** | **28.69** | **28.93** | **30.43** | **32.55** | **33.27** |
| CGJ sample1 | 30.31 | 19.86 | 12.75 | 6.81 | 8.94 | 6.64 | 28.52 | 20.60 | 12.25 | 5.69 | 14.94 | 6.21 | 9.70 |
| CGJ sample2 | 31.38 | 19.63 | 10.02 | 5.03 | 9.13 | 6.18 | 29.64 | 21.91 | 12.13 | 5.67 | 14.49 | 7.43 | 9.70 |
| CGJ sample3 | 32.52 | 20.21 | 9.03 | 5.93 | 8.77 | 8.12 | 29.51 | 20.98 | 10.87 | 5.29 | 14.32 | 8.80 | 9.72 |
| CGJ sample4 | 32.39 | 19.89 | 14.56 | 6.56 | 8.51 | 7.29 | 21.70 | 21.06 | 11.65 | 5.40 | 14.61 | 8.49 | 10.31 |
| CGJ sample5 | 27.77 | 14.70 | 14.40 | 6.30 | 8.79 | 6.56 | 25.75 | 21.42 | 10.34 | 5.09 | 13.95 | 9.23 | 10.05 |
| CGJ sample6 | 34.02 | 17.58 | 13.92 | 6.58 | 8.51 | 5.72 | 24.27 | 20.27 | 9.56 | 5.46 | 14.24 | 9.02 | 9.58 |
| CGJ sample7 | 30.21 | 13.52 | 13.27 | 6.35 | 8.48 | 7.03 | 24.22 | 17.98 | 12.29 | 5.63 | 10.82 | 9.02 | 5.81 |
| CGJ sample8 | 29.39 | 16.20 | 14.15 | 6.93 | 8.72 | 6.62 | 22.68 | 16.40 | 11.27 | 5.57 | 14.86 | 7.30 | 8.22 |
| CGJ sample9 | 29.90 | 15.09 | 14.63 | 6.16 | 8.67 | 6.77 | 27.91 | 20.47 | 11.71 | 5.30 | 16.40 | 6.16 | 9.24 |
| CGJ concentrate1 | 212.00 | 121.40 | 85.42 | 43.87 | 57.63 | 48.79 | 133.27 | 112.41 | 60.03 | 42.78 | 129.75 | 54.07 | 64.48 |
| CGJ concentrate2 | 190.18 | 115.24 | 81.66 | 46.41 | 51.56 | 53.94 | 126.55 | 114.03 | 63.05 | 42.82 | 124.76 | 58.28 | 65.07 |
| CGJ concentrate3 | 212.51 | 129.80 | 78.55 | 44.72 | 60.23 | 53.37 | 125.85 | 116.45 | 60.49 | 41.64 | 127.66 | 54.77 | 65.99 |

RT^a^, retention time. All data are expressed in μg/mL. Dp, delphinidin, Cy, cyanidin, Pt, petunidin, Mv, malvidin, Pn, peonidin, Pt, petunidin, G, glucoside or other hexoside, Ac, acetyl, Co, coumaroyl.

**Table S8.** Content of tentatively identified flavonols in Concord Grape Juice (CGJ).

|  | **c1** | **c2** | **c3** | **c4** | **c5** |
| --- | --- | --- | --- | --- | --- |
| **Compound** | **Rut** | **My-G** | **Q-G** | **Q-GR** | **Q** |
| **RT^a^ (min)** | **10.79** | **20.98** | **25.02** | **25.51** | **43.14** |
| CGJ sample1 | 43.90 | 6.65 | 11.69 | 7.85 | 1.30 |
| CGJ sample2 | 41.64 | 7.94 | 9.62 | 5.42 | 1.41 |
| CGJ sample3 | 40.61 | 11.22 | 9.23 | 10.47 | 1.74 |
| CGJ sample4 | 41.93 | 13.83 | 9.57 | 6.30 | 1.94 |
| CGJ sample5 | 33.59 | 12.74 | 8.07 | 11.63 | 1.62 |
| CGJ sample6 | 33.74 | 13.70 | 7.79 | 12.00 | 1.70 |
| CGJ sample7 | 39.99 | 13.19 | 7.09 | 12.64 | 1.58 |
| CGJ sample8 | 32.34 | 12.50 | 12.28 | 12.03 | 0.96 |
| CGJ sample9 | 34.08 | 7.58 | 10.91 | 8.90 | 1.48 |
| CGJ concentrate1 | 220.14 | 64.89 | 70.36 | 71.73 | 26.95 |
| CGJ concentrate2 | 230.92 | 78.18 | 74.27 | 76.94 | 27.01 |
| CGJ concentrate3 | 213.78 | 83.52 | 69.60 | 68.78 | 26.79 |

RT^a^, retention time. All data are expressed in μg/mL. My, myricetin, Q, quercetin, G, glucoside or other hexoside, Co, coumaroyl, GR, glucuronoyl.

**Table S9.** Content of targeted phenolic compounds in Concord Grape Juice (CGJ).

| **Compound** | **CGJ sample1** | **CGJ sample2** | **CGJ sample3** | **CGJ sample4** | **CGJ sample5** | **CGJ sample6** | **CGJ sample7** | **CGJ sample8** | **CGJ sample9** | **CGJ concentrate1** | **CGJ concentrate2** | **CGJ concentrate3** |
| --- | --- | --- | --- | --- | --- | --- | --- | --- | --- | --- | --- | --- |
| 3-hydroxytyrosol | 0.013 | 0.015 | 0.013 | 0.016 | 0.013 | 0.015 | 0.017 | 0.016 | 0.020 | 0.161 | 0.138 | 0.147 |
| isochlorogenic acid | 0.007 | 0.008 | 0.007 | 0.007 | 0.007 | 0.008 | 0.018 | 0.010 | 0.005 | 0.024 | 0.030 | 0.029 |
| 3,4-dihydroxybenzoic acid | 0.659 | 0.624 | 0.644 | 0.599 | 0.651 | 0.630 | 0.624 | 0.626 | 0.634 | 4.185 | 4.415 | 4.426 |
| 4-methyl gallic acid | 0.272 | 0.268 | 0.283 | 0.262 | 0.287 | 0.272 | 0.291 | 0.269 | 0.295 | 1.333 | 1.282 | 1.338 |
| catechin | 2.357 | 2.305 | 2.288 | 2.171 | 2.300 | 2.297 | 2.106 | 2.217 | 2.107 | 28.284 | 27.790 | 27.979 |
| procyanidin B2 | 3.876 | 3.868 | 3.907 | 3.465 | 3.647 | 3.732 | 3.616 | 3.453 | 3.613 | 20.149 | 18.700 | 19.695 |
| epicatechin | 1.905 | 1.838 | 1.797 | 1.835 | 1.919 | 2.087 | 2.019 | 1.943 | 1.864 | 27.687 | 27.346 | 27.589 |
| 3-hydroxybenzoic acid | 0.101 | 0.092 | 0.105 | 0.099 | 0.097 | 0.096 | 0.095 | 0.093 | 0.097 | 0.962 | 0.994 | 1.027 |
| caffeic acid | 7.394 | 7.168 | 7.266 | 6.763 | 6.975 | 6.954 | 6.666 | 6.663 | 6.668 | 57.554 | 57.036 | 57.856 |
| 4-hydroxybenzoic acid | 0.374 | 0.357 | 0.366 | 0.379 | 0.367 | 0.348 | 0.339 | 0.358 | 0.353 | 2.806 | 2.796 | 2.907 |
| vanillic acid | 0.096 | 0.092 | 0.093 | 0.086 | 0.087 | 0.091 | 0.102 | 0.096 | 0.088 | 0.610 | 0.663 | 0.657 |
| dihydromyricetin | 0.110 | 0.118 | 0.118 | 0.116 | 0.125 | 0.112 | 0.167 | 0.121 | 0.111 | 0.654 | 0.667 | 0.663 |
| syringic acid | 0.375 | 0.332 | 0.328 | 0.312 | 0.333 | 0.370 | 0.343 | 0.389 | 0.391 | 1.893 | 1.990 | 2.052 |
| resveratrol-3-glycoside | 0.569 | 0.560 | 0.571 | 0.515 | 0.593 | 0.521 | 0.516 | 0.530 | 0.534 | 4.462 | 4.391 | 4.453 |
| dihydroferulic acid | 0.058 | 0.039 | 0.052 | 0.070 | 0.051 | 0.051 | 0.052 | 0.062 | 0.049 | 0.506 | 0.453 | 0.511 |
| sinapic acid | 5.559 | 5.530 | 5.260 | 5.275 | 5.499 | 5.404 | 5.007 | 5.149 | 5.136 | 0.347 | 0.339 | 0.338 |
| taxifolin | 0.046 | 0.045 | 0.046 | 0.043 | 0.049 | 0.045 | 0.041 | 0.042 | 0.046 | 0.010 | 0.016 | 0.009 |
| ferulic acid | 0.001 | 0.000 | 0.002 | 0.002 | N.D. | 0.001 | N.D. | 0.001 | 0.003 | 3.555 | 3.506 | 3.436 |
| 3-hydroxycinnamic acid | 0.431 | 0.451 | 0.471 | 0.382 | 0.439 | 0.418 | 0.457 | 0.387 | 0.420 | 50.375 | 50.846 | 50.636 |
| phenylacetic acid | 0.100 | 0.101 | 0.118 | 0.102 | 0.113 | 0.112 | 0.099 | 0.118 | 0.087 | 0.255 | 0.396 | 0.361 |
| *trans*-2-hydroxycinnamic acid | N.D. | 9.273 | 9.484 | 9.448 | 9.086 | 9.352 | 9.008 | 8.748 | 9.063 | 73.955 | 73.543 | 72.070 |

All data in μg/mL

**Table S10**. Concord Grape Juice (CGJ) stability test collection time points.

| Time point | Collect date | Duration time |
| --- | --- | --- |
| 0 | 05/26/2021 | 0 |
| 1 | 06/02/2021 | 1 week |
| 2 | 06/09/2021 | 2 weeks |
| 3 | 07/16/2021 | 1 month |
| 4 | 08/16/2021 | 2 months |
| 5 | 09/16/2021 | 3 months |
| 6 | 10/16/2021 | 4 months |
| 7 | 11/16/2021 | 5 months |
| 8 | 12/16/2021 | 6 months |
| 9 | 02/16/2022 | 8 months |
| 10 | 04/16/2022 | 10 months |
| 11 | 06/16/2022 | 12 months |

**Table S11. Concord Grape Juice (**CGJ) preliminary stability study data

|  | **Anthocyanidin** | **Flavonols** | **Phenolic compounds** |
| --- | --- | --- | --- |
| month 0 | 181.26 ± 4.03 | 71.57 ± 2.65 | 32.61 ± 1.15 |
| month 4 | 177.39 ± 6.94 | 69.82 ± 0.64 | 31.40 ± 1.20 |
| month 6 | 152.64 ± 2.21 | 66.05 ± 0.80 | 30.84 ± 0.22 |

All data in μg/mL

**Figure S1.** Detected content of epicatechin, procyanidin B2, catechin and gallic acid in GSE capsule using extraction solvent of 70% methanol acidified using 1% formic acid, and water with 1% formic acid.
